# Fifty Years of Wildlife Diseases in Europe: A Citation Database Meta-Analysis

**DOI:** 10.3390/vetsci9110629

**Published:** 2022-11-11

**Authors:** Rachele Vada, Stefania Zanet, Ezio Ferroglio

**Affiliations:** Department of Veterinary Sciences, Università Degli Studi di Torino, Largo Paolo Braccini, 2, Grugliasco, 10095 Torino, Italy

**Keywords:** zoonoses, wild animals, scientific databases, trend analysis, publication trends

## Abstract

**Simple Summary:**

In the past decades, wildlife has been progressively recognized as relevant for the circulation of pathogens affecting not only wild species but also humans and domestic animals. Currently, there is no clear picture regarding which pathogens have been the subject of research over the years and how the investigation has evolved over time. Such information would be useful to guide future steps for wildlife disease management. In this paper, we aimed at answering this question by going through the outputs of a citation database, Web of Science, in terms of the number of wildlife disease publications, and the frequency of disease related MeSHs. Overall, the scientific interest increased over time, with a peak in the last 20 years. The focus of research changed over time and shifted to contemporary hot topics, such as zoonoses, conservation-related diseases and emergent diseases. The increasing complexity of diseases related to wildlife is an aspect that should be kept in mind when drafting surveillance and control plans.

**Abstract:**

Although wildlife has progressively been recognized as a booster for the spillover of pathogens to humans and other wild and domestic animals, the interest of scientists in this topic has not been constant over time and uniform in its targets. Epidemiological outbreaks and technological development have contributed to this. Through the analysis of the number of publications from a citation database, we aimed to obtain an indicator of the scientific community’s interest towards wildlife diseases over the years. Using Web of Science, bibliographic searches were performed by adding to the basic search string “Wildlife AND Disease” terms detailing topics such as aspect investigated, pathogen type, aetiologic group and species group. For each host species group, the 100 most frequent Medical Subject Headings (MeSHs) related to specific diseases in each decade were extracted. The scientific production regarding wildlife diseases has increased 3.7 times the relative proportion of publications on diseases during the last 50 years, focusing mainly on zoonotic or epizootic pathogens, and with a sharp growth in contemporary hot topics. Wildlife disease scenarios are complex and challenging to approach. Knowing the trends in the scientific interest in the past decades may pose a guide to direct future steps and actions in several fields, from public health to ecosystem management.

## 1. Introduction

With the progressive consolidation of the One Health concept, the role of wildlife as a reservoir and spillover denominator has gathered increasing attention [1]. In shared ecosystems, the health of humans, animals and the environment becomes interconnected, and pathogens coevolve with populations [2]. For millennia, pathogens jumped the species barrier from animals to humans and vice versa. In the past 20 years, 60.3% of emerging infectious diseases affecting humans originated from animals, and the majority of these (71.8%) came from wildlife [3]. Wildlife disease surveillance systems and control programs play a central role in preventing and controlling the spillover of pathogens at the human–livestock–wildlife interface [4]. One of the first examples is the rabies vaccine campaign in wild canids [5,6].

Wildlife is without any doubt a key factor for the circulation of zoonotic or epizootic pathogens of great relevance, such as *Echinococcus multilocularis* [7] or the avian influenza virus [8]. Additionally, wildlife can boost the circulation of vectors: *Hyalomma* ticks were introduced to Spain through the migratory routes of wild birds from Africa and, in recent years, have been responsible for introducing the Crimea–Congo hemorrhagic fever virus to the Iberian peninsula [9]. Likewise, global warming and climatic changes have favored vectors and diseases to expand to previously unsuitable areas [10]: *Leishmania infantum* has spread northwards in the past decades and its expansion is bound to continue [11]. Recently, interest in wildlife diseases has also included pathogens that pose a risk to the conservation of biodiversity [12].

Despite certain limits of accuracy, publication and citation counts can be considered valuable indicators of scientific research interest and its fluctuations over time [13]. By describing and quantifying the scientific research carried out on wildlife diseases, important information can be obtained to define future research topics and research tools.

The purpose of the study was to analyze the trend of publications on wildlife diseases in Europe in the past 50 years, by defining the main research targets and how they changed over time in terms of pathogens and target host species. This analysis compared the relative importance attributed to diseases and host species by various stakeholders within the public health, veterinary and environmental sectors.

## 2. Materials and Methods

The number of articles published about wildlife diseases makes a systematic review of each paper impractical and uninformative.

A more general approach was adopted. We explored the query outputs using the specific analysis feature provided by the citation database (https://support.clarivate.com/ScientificandAcademicResearch/s/article/Web-of-Science-Steps-to-analyze-results?language=en_US, accessed on 14 September 2022). The query addressed all databases and collections included in Web of Science and each specific search string searched pertinent papers by correspondence between the search string itself and the “Topic” of each paper (which in Web of Science includes title, abstract and key words). The bibliographic search was performed between November and October 2021. The first query algorithm was: “wildlife” AND “disease”. We ran the most general and simple search string to ensure a solid research baseline. Exploratory trials with more specific search strings showed no difference in temporal trends (data not shown). The query was then refined for countries of the European continent (thus including all papers whose authors are affiliated to an organism or institution belonging to countries of the European continent). No temporal limitations were applied to publication year.

Subsequent queries added terms one at a time to the algorithm with the “AND” operator. Additional terms belonged to four clusters: (i) disease aspect investigated, (ii) disease type, (iii) disease agent type, (iv) target species group and (v) publication type. Terms are listed in Table 1 and the query links are reported in Supplementary Table S1.

For each query, we retrieved the number of articles published per year and grouped them by decade for subsequent analysis. We performed a linear regression between the years and the logarithm of the number of publications to obtain the regression coefficient as an indicator for the increment in the number of papers over time. The regression’s R^2^ pointed out how much of the increase was attributable to the progress of time. Moreover, the cumulative H index was extracted for each search string and normalized over the total number of publications. This parameter was also used as an indicator of interest on a specific topic.

Finally, for each group of species (the full list is reported in Table S1), we downloaded the first 100 MeSHs (Medical Subject Headings) ranked by frequency for each decade. After an exploratory overview to spot any recurrent patterns, terms referring to a pathogen or disease were extracted. The frequencies of those referring to the same disease were merged. Tick-Borne Diseases were also merged. Consequently, a classification of the most frequent MeSHs related to a specific disease was obtained, divided per species group and decade. Eventually, the classification was graphically represented and analyzed.

## 3. Results

Since 1940, a total of 63,170 publications concerning wildlife diseases have been produced globally. Of these, 21,254 (33.65%) are by Europe-based authors, with the first papers published in 1972. Thus, the last 50 years (1972–2021) was the analyzed period. The results for all queries are reported in Supplementary Table S2. Comparing with publications about diseases in general, “wildlife” extracted 0.154% of publications in 1972 and 0.574% in 2021, increasing the proportion by 3.7 times. The country operativity in wildlife disease research was greater (43%) in Northern European countries (according to the United Nations Geoscheme, www.unstats.un.org, accessed on 20 September 2022). All additional terms used in this study returned a positive regression coefficient. The R^2^ showed a strong link between the year and the number of publications, fluctuating between 0.85 and 0.92. The seven exceptions are the outputs of the searches with: vector-borne (R^2^ = 0.54), prion (R^2^ = 0.79), rodents (R^2^ = 0.65), insectivores (R^2^ = 0.44), bat (R^2^ = 0.70), conference (R^2^ = 0.55) and books (R^2^ = 0.40). In those cases, the increase in the number of publications is only partially explicable by the progression of the years.

**Figure 1:**
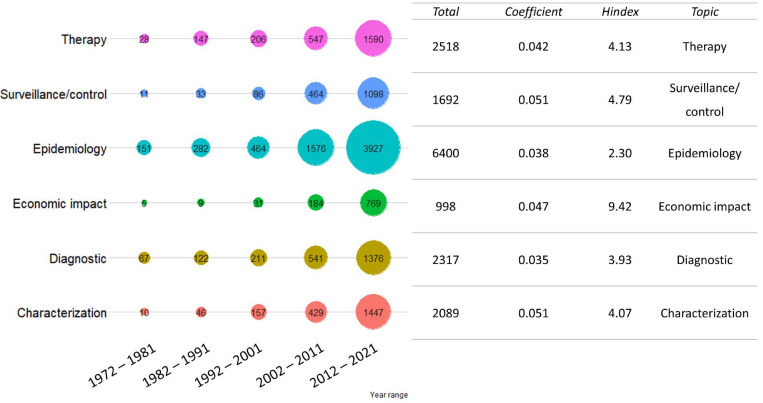
Publication trend for “aspect of disease investigated” cluster. The total number of publications was grouped by ten-year period. For each disease aspect investigated, the total number of publications in the last 50 years, regression coefficient (as growth rate) and cumulative H index divided by the number of publications are shown on the right. Colors are not informative.

In the “aspect of disease investigated” cluster, the highest number of publications was returned by the term “epidemiology” (6400 publications), followed by “therapy” (2518) and “diagnostic” (2317). However, the higher coefficients of “surveillance” (0.051) and “economic impact” (0.047) reflect a sharper increase for related publications in the last decades. On the contrary, economic impact collected the highest relative H index. The results are presented in Figure 1.

**Figure 2:**
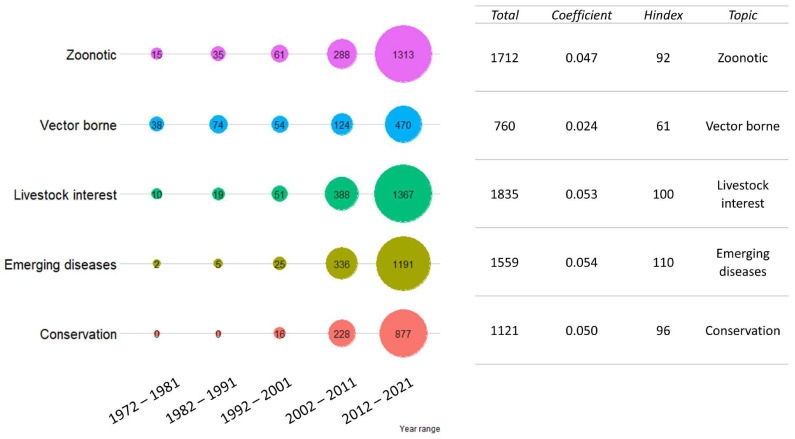
Publication trend for the “disease type” cluster. The total number of publications was grouped by ten-year period. For each disease type, the total number of publications in the last 50 years, regression coefficient (as growth rate) and cumulative H index divided by the number of publications are shown on the right. Colors are not informative.

Although “zoonotic” and “livestock” are the terms that returned the highest numbers of publications (1712 and 1835, respectively), the outputs of “biodiversity conservation” and “emerging diseases” presented a strong increase in the last decades, with two of the highest regression coefficients (0.050 and 0.054, respectively). “Vector-borne” returned a more constant growth (0.024), while “conservation” returned the highest relative H index. The results are presented in Figure 2.

**Figure 3:**
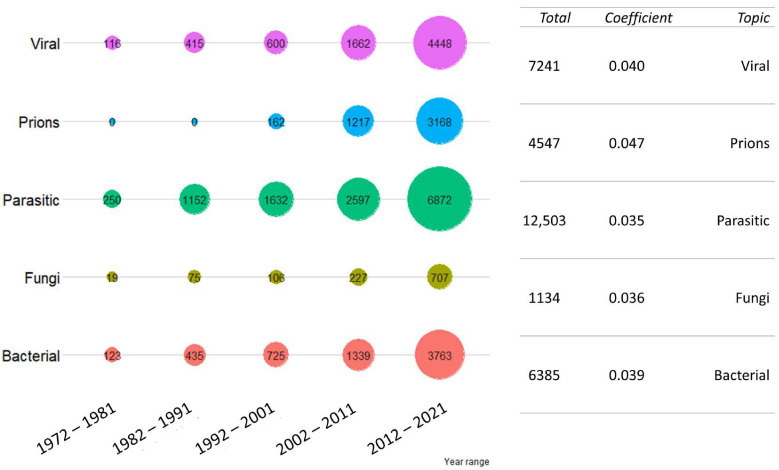
Publication trend for the “disease agent type” cluster. The total number of publications was grouped by ten-year period. For each disease agent type investigated, the total number of publications in the last 50 years and regression coefficient (as growth rate) are shown on the right. Colors are not informative.

Parasites are the etiological group which returned the highest number of publications, double the number of publications on viruses and bacteria (1250 total publications). Despite this, viruses and prions returned the highest regression coefficient (0.040 and 0.047, respectively). The H index was not produced for parasites, due to the number of publications (Web of Science returns such values when the number of outputs is less than 10,000); therefore, comparison of this parameter was not possible. The results are presented in the Figure 3.

**Figure 4:**
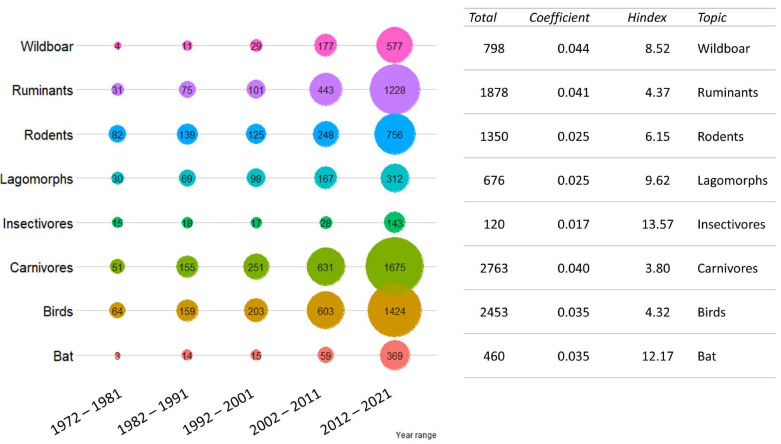
Publication trend for the target species group investigated. The total number of publications was grouped by ten-year period. For each target species group investigated, the total number of publications in the last 50 years, regression coefficient (as growth rate) and cumulative H index divided by the number of publications are shown on the right. Colors are not informative.

Among the queries for species groups, “carnivores” was the term that returned the highest number of publications (2763) and “birds” the second highest (2453). The principal increase in publication numbers regarded “wild boar” and “wild ruminants” (0.045 and 0.041, respectively), while “lagomorphs” and “rodents” returned some of the lowest (0.025 for both), maintaining a more constant publication rate over the years. The results are presented in the Figure 4.

**Figure 5:**
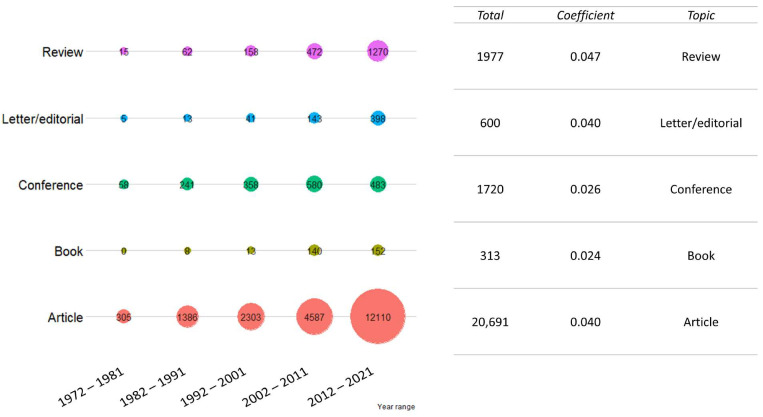
Publication trend for the “publication type” cluster. The total number of publications was grouped by ten-year period. For each publication type investigated, the total number of publications in the last 50 years and regression coefficient (as growth rate) are shown on the right. Colors are not informative.

Articles are the most common type of publication (20,691). Despite this, reviews have had the sharpest increase in the last decades (coefficient 0.047), while book production remained almost constant (0.023). Conferences were the second most common type of publication in the 1970s (58), although the scenario changed in the following decades. The results are presented in Figure 5.

**Figure 6:**
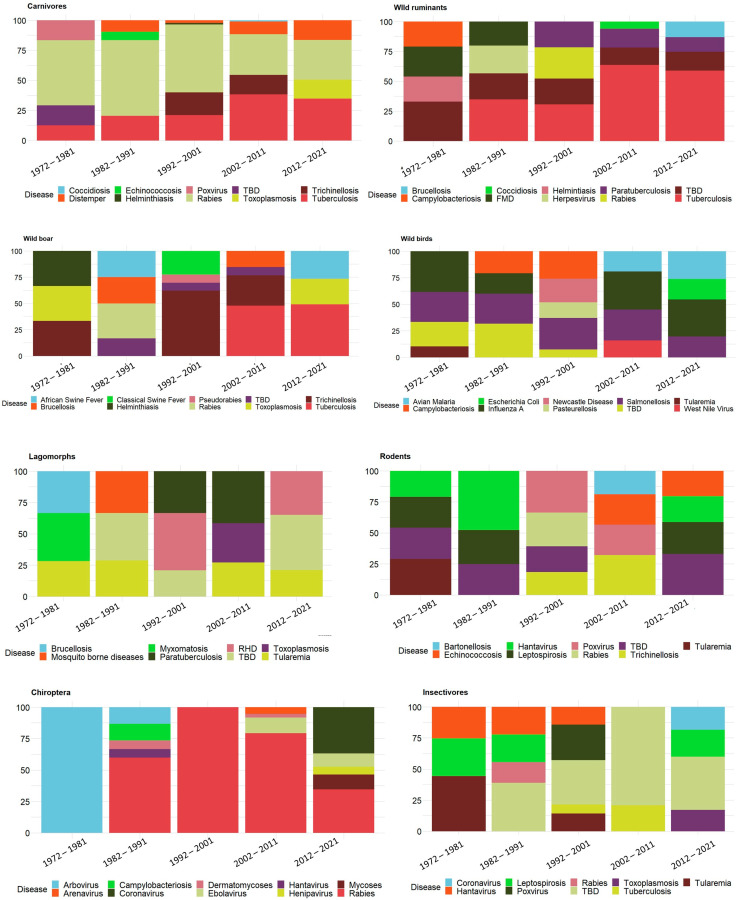
For each species group and decade, the first 10 most frequent (per species group, considering all decades) disease-related MeSH terms are represented with their relative frequency to the decade. Colors vary according to the species group (legend below each group). X axis: decade; Y axis: percentage of relative frequency. TBD = tick-borne disease. FMD = foot and mouth disease. RHD = rabbit hemorrhagic fever.

The complete results of the MeSH analysis are presented in Supplementary Table S3, while the graph in Figure 6 presents the first 10 MeSH terms per species group. Tick-borne diseases present high frequencies in all mammals, while tuberculosis increased in susceptible species. A similar trend can be spotted for zoonoses such as trichinellosis, echinococcosis and toxoplasmosis. While wild ruminants present a polarization towards fewer disease related MeSHs, the wild boar search showed an increased complexity in the number of pathogens. In carnivores, rabies decreased its frequency over the decades. Lagomorphs presented a high variety of different diseases, with tularemia being among the most frequently reported since the 1970s and rabbit hemorrhagic disease since the 1990s. In rodents, high frequencies of hantaviruses and leptospirosis are present in all decades. Chiropterans showed a predominance for viral diseases. As a constant over time, avian influenza dominated the research interest in wild birds, followed by salmonellosis and campylobacteriosis and, with lower frequencies, by the West Nile virus and malaria. Finally, the high complexity of pathogens presented by insectivores could be biased by the small number of publications, which leads almost all MeSH terms to enter the first 100.

## 4. Discussion

The scientific production regarding wildlife diseases has undergone a transversal increase on all the main research topics during the last 50 years, with a predominant interest in epidemiological studies and a more recent sharp growth in fields that are also contemporary hot topics, such as emerging diseases, pathogens that threaten biodiversity or prion-borne diseases. The fluctuations in terms of the number of publications for each species group and disease reflect the changes through the last decades in the health concerns regarding wild and domestic animals.

The increase in scientific activity is a trait that accompanies all aspects of research, and this trend affects wildlife disease publications as well. About a third of worldwide papers published on the topic comes from European countries, with a major proportion from northern countries. This scenario may reflect the idea that the interest in research, and in one topic rather than another, is dependent on economic wealth [14]. We can therefore suppose that wildlife is still not a priority research field worldwide. However, the increase in the number of publications, especially in the last two decades, may be an indicator of the rising awareness of the crucial role that wild species play in the circulation of pathogens of zoonotic or economic interest [1]. Moreover, it must be kept in consideration that the attribution of a paper to a country by the Web Of Science platform is based on the authors’ affiliations, rather than on the geographic setting of the study. Therefore, it would be more accurate to speak of country operativity rather than of research activity in the country.

Epidemiology has always been recognized as a crucial part of the control efforts against transmissible diseases, and papers that involve this aspect have dominated the scene in the last decades. However, the sharp increase in publications including surveillance and control may be considered an indicator that countries are starting to value the importance of wildlife within a comprehensive investigation approach for disease management [15]. Parallelly, the economic impact of a spillover from wildlife is also being recognized as having potentially important outcomes, as it has been remarked upon in the literature [16]. Pathogens that affect human or livestock health have always received interest, as it emerged in both the temporal trends and MeSH frequency of the related query outputs. In recent years, the peak in publications that include diseases threatening biodiversity broadens the spectrum of One Health even more; its focus has come to include not only public health and livestock, but also ecosystem conservation, in light of the role of pathogens as a threat to the survival of endangered species [17] and that of biodiversity as a determinant factor for the reduction in disease rates [18]. Climate change and global warming effects were reflected in publication trends; the emerging diseases search term returns an increasing number of publications in Europe, and several studies have already detected the spread of related pathogens to new territories [11].

An analysis focusing only on long-term studies on wildlife diseases [19] has shown similar results to the current work regarding the top-ranking target diseases. On the contrary, the work by Barroso and colleagues [19] arrived at different conclusions when grouping pathogens into viruses, bacteria and parasites, the latter being only the third most popular topic. In the current work, which is not limited to long-term research projects, papers involving parasitic diseases are the most frequently published. Despite the highest frequency of parasitology-targeting papers, MeSHs showed a predominance of bacterial and viral diseases. It is legitimate to suppose that, despite the high number of papers, the parasitic world is extremely complex and only a few singular parasitic diseases appeared in top frequencies of MeSH terms. For example, the choice to group all tick-borne diseases has allowed us to highlight the importance of ticks as pathogen vectors.

An OIE wildlife health survey report published in 2021 [20] listed the main wild species that should be targeted by surveillance programs involving wildlife, according to the veterinary authorities of the OIE. The priority pattern emerging from the OIE survey places birds as the top-ranking taxa, while our meta-analysis ranked carnivores as the most frequent species group, followed by birds. The avian influenza A virus is most likely the cause behind this inconsistency between the two analytical strategies. Avian influenza is the principal pathogen that appears in publications regarding wild birds, with a constant effort over time. Moreover, in the same OIE report, it was selected as the principal disease on which wildlife surveillance was needed, undoubtedly because of the recent outbreaks and their consequences [21], which are of particular relevance considering the OIE mission and scopes. This could be an indicator of the fact that, despite being carnivore carriers of significant pathogens that can affect livestock and humans as well (i.e., the increasing publication trend on tuberculosis and echinococcosis), their role is resized, and priority is given to other diseases. Moreover, the original interest towards carnivores was mainly focused on rabies, until the vaccination campaigns reduced its worldwide burden. The intense and recently increased investigation on the distemper virus is also noteworthy, confirming the trend of interest in pathogens that do not concern human or livestock health, but have significant impacts on conservation [17].

The importance of wild ungulates is commonly recognized and involves diseases of great concern worldwide, such as tuberculosis or brucellosis [4]. Moreover, the explosion of prion-related publications was strictly related to the outbreaks of bovine spongiform encephalopathy and the recent emergence of chronic wasting disease [22]. The MeSH “population density” was also quite frequent for wild ruminants. This topic has been in the spotlight in recent years, as many wildlife diseases are strictly dependent on density, and the latter is a determinant factor for disease control [23]. In the case of wild boar, it is remarkable how the number of publications targeting this single species are equal to one third of the publications of the entire carnivore group and to half of those of all wild ruminants together. Considering that the complexity of pathogens investigated is increasing alongside the number of studies, it is clear how this species was progressively recognized as an important host of several relevant diseases. These diseases related to wild boar have changed over time. Considering the variety of diseases for which the wild boar is epidemiologically relevant [24], our data confirm this species as one of the main species of research interest in Europe. This relevance is also attributable to its extensive use as wild game meat. According to FAO data, wild boar is the most frequently consumed wild ungulate across Europe, and the implications in terms of public health make this species of particular interest for food-borne diseases [25].

As for chiropterans, the high importance given by the OIE and the relatively low number of publications may be due to the fact that the discovery of such species as carriers of high-impact zoonotic pathogens in Europe was mostly recent [26]. The diseases identified by our MeSH search are also identified by OIE members as emergent diseases with a high risk of spillover from wildlife to humans and that should be targeted with surveillance programs [20]. Current research on bats targets diseases with high visibility, such as hantaviruses, ebolaviruses or coronaviruses. On the other hand, rodents, which are long- known as carriers of zoonotic pathogens [27], have generally had a constant trend in publications. The diseases related to rodents are numerous and varied, as shown in the present study, and are of great interest for human health. Outbreaks of rabbit hemorrhagic fever, myxomatosis or tularemia were already well-known in lagomorphs at the beginning of our study period [28], and interest towards this group of species was steadier compared to others. A decline in the number of publications on myxomatosis mirrored a rise in the interest toward the rabbit hemorrhagic disease virus, a pathogen with significant implications for conservation [28]. Being carriers of zoonotic and conservation-related diseases, and recently discovered as carriers of emerging pathogens such as *L. infantum* [29], it is surprising how lagomorphs are still a marginal focus in European wildlife disease research. Insectivores, as well as rodents, are typical wildlife that inhabits peri-urban (or even urban) environments and thus are a reservoir of tick-borne diseases, as already suggested [30]. They can represent a potentially important source of infection because of the geographical proximity to humans and the high burden of feeding ticks [30].

## 5. Conclusions

As wildlife is an essential component in the epidemiology of many, if not most, zoonoses, it should be considered in risk analysis frameworks and in disease surveillance systems. The scientific activity does not always match the priorities identified by stakeholders, and the wildlife disease scenario is becoming complex rather than polarized, a situation that should be well considered when addressing surveillance plans. This should also be recognized in relation to target host species, as neglected ones are of interest as important reservoirs of relevant diseases. Following the recent trend recognizing the importance of a One Health approach, we may expect increasing interest in wildlife disease surveillance and emergent pathogens. Likewise, zoonoses will maintain relevance, as food-borne pathogens for game animals, and viruses for other species. We may also expect an increase in complexity, both in terms of pathogens and in terms of target species.

The geographic extent (limited to Europe) and the methodologies of the work make these results subject to improvement. Of course, this analysis left aside many groups of hosts (reptiles, amphibians, fish and marine mammals) that would be worth considering. A transboundary characterization of disease epidemiology is fundamental in a globalized world, where pathogens move together with animals and people. A similar analysis carried out on a global scale and focused on different macro-regions may provide information useful for a One Health approach to disease control.

## Figures and Tables

**Table 1:** Scheme of the additional term clusters.

Disease Aspect Investigated	
Epidemiology	Therapy
Diagnostic	Surveillance
Identification	Economic impact
**Disease Type**	
Vector-borne	Livestock interest
Zoonotic	Biodiversity conservation
Emerging disease	Infection
**Disease Agent Type**	
Bacteria	Parasite
Virus	Fungi
Prions	
**Target Species Group**	
Carnivores	Lagomorphs
Wild ruminants	Chiroptera
Wild boar	Insectivores
Rodents	Birds
**Publication Type**	
Article	Letter/editorial
Review	Conference
Book	

## Data Availability

Data are contained within the article or Supplementary Materials.

## Supplementary Materials

The following supporting information can be downloaded at: https://www.mdpi.com/article/10.3390/vetsci9110629/s1; Table S1: link to the queries; Table S2: number of outputs for each query; Table S3: ranking of the 100 most frequent disease-related MeSH terms per species group and decade.
